# Chemical Composition Variation in Essential Oil and Their Correlation with Climate Factors in Chinese Prickly Ash Peels (*Zanthoxylum armatum* DC.) from Different Habitats

**DOI:** 10.3390/molecules29061343

**Published:** 2024-03-18

**Authors:** Qianqian Qian, Zhihang Zhuo, Yaqin Peng, Danping Xu

**Affiliations:** College of Life Science, China West Normal University, Nanchong 637002, China; qianqqqchen@foxmail.com (Q.Q.); zhuozhihang@foxmail.com (Z.Z.); pengyaqin2023@foxmail.com (Y.P.)

**Keywords:** *Zanthoxylum armatum* DC., essential oil, climate factors

## Abstract

Essential oils are secondary metabolites in plants with a variety of biological activities. The flavor and quality of *Zanthoxylum armatum* DC. are mainly determined by the essential oil components in the Chinese prickly ash peels. In this study, the correlation between climate change in different regions and the content of essential oils of *Z. armatum* was investigated using gas chromatography–mass spectrometry (GC/MS) and multivariate statistical analysis. The Z1–24 refers to 24 batches of samples from different habitats. A total of 145 essential oils were detected in 24 batches of samples, with the highest number of terpene species and the highest content of alcohol. The relative odor activity (ROAV) values identified nine main flavor compounds affecting the odor of *Z. armatum*. Linalool, decanal, and d-limonene were the most critical main flavor compounds, giving *Z. armatum* a spicy, floral, oily, and fruity odor. The results of hierarchical cluster analysis (HCA) and principal component analysis (PCA) classified Z5 into a separate group, Z2 and Z7 were clustered into one group, and the rest of the samples were classified into another group. Correlation analysis and path analysis showed that temperature and precipitation were the main climatic factors affecting essential oils. Comparisons can be made with other plants in the genus Zanthoxylum to analyze differences in essential oil type and content. This study contributes to the identification of *Z. armatum* quality, promotes the accumulation of theories on the effects of climatic factors on essential oils, and enriches the site selection and breeding of *Z. armatum* under similar climatic conditions.

## 1. Introduction

*Zanthoxylum armatum* DC. is a small tree or shrub in the genus *Zanthoxylum* L. of the family Rutaceae. There are more than 250 species of the genus *Zanthoxylum* L. in the world, including 45 species and 13 varieties in China [1]. Worldwide, the genus is found mainly in Asia, including Central, South, Southeast, and East Asia, but also in Himalayas, Americas, and Africa [2]. In China, *Z. armatum* are mainly distributed in the southwestern region, such as Sichuan, Chongqing, Yunnan, and Guangxi [3]. *Z. armatum* peels are rich in amides, essential oils, flavonoids, and coumarins, which have a pungent flavor and medicinal value [4]. *Z. armatum* peels, as an herbal medicine, can relieve fever, appetite, toothache, rheumatism, diabetes, etc. [2,5]. It is commonly used as an edible spice for making kimchi, hot pot seasoning, and cooking traditional Chinese dishes and is one of the “Eight Major Seasonings” in China. [1]. Being a flavoring product, *Z. armatum* needs to be controlled in terms of quality. Essential oil is the main substance that affects the quality of *Z. armatum* [6], and the aroma of *Z. armatum* peels is mainly determined by volatile components [1]. Essential oil is a group of secondary metabolites obtained from plants, such as alcohols, ketones, aldehydes, and esters, which are usually found in aromatic plants [7]. There are about 3000 known types of essential oils, which are commonly used in the pharmaceutical, food, and cosmetic industries, among others [7,8]. It is also biologically active, capable of being antioxidant, antibacterial, anti-inflammatory, and analgesic [9]. In previous studies, Zheng et al. [10] analyzed the chemical composition of essential oils and their relationship with climatic factors in peel samples of *Zanthoxylum bungeanum* Maxim. For the present study, the species *Z. armatum* was selected. Phuyal et al. [5] investigated the essential oil composition of *Z. armatum* leaves from Nepal by GC/MS and studied the effect of altitude and soil chemical composition on their content. In this study, on the other hand, seven climatic data were selected from the perspective of climatic factors to analyze their relationship with major flavor compounds. A total of 24 samples from *Z. armatum*-producing areas were selected, so the sample size is sufficiently large.

The composition and content of essential oils are affected by various factors, such as variety [11], origin [10], harvesting time [12], and storage conditions [13]. It is worth noting that environmental conditions can greatly affect the composition and content of essential oils [14]. Essential oils are secondary metabolites in plants [15], which can help plants resist and adapt to external environmental changes during their growth and development, so their content and composition will change significantly with environmental changes [10]. The effect of climatic factors on essential oils was also analyzed in the literature in previous studies. Yasar et al. [16] found that thymol and carvacrol compositions of the above-ground parts of different Origanum hybrids were either increased or decreased under greenhouse conditions with different levels of CO_2_. Karalija et al. [14] concluded that due to the significant difference in average temperature and humidity between the coastal and continental areas of Croatia, there was a significant difference in the thujones content of *Salvia officinalis* L. grown in this location. *Cirsium arvense* L. plants from four districts were collected and analyzed for essential oil composition and content by GC/MS by Amiri et al. [17]. Climate and soil characteristics were found to have a significant effect.

Among the available studies on the essential oils of *Z. armatum* include the study of their antioxidant properties [18], in vivo evaluation of antiasthmatic activity [19], and chemical constituents [20]. Previous studies focused on functional and compositional studies. The objective of this study was to identify the major compounds that affect the flavor of *Z. armatum*. The effect of climatic factors on the content of these major flavor compounds was also analyzed. A large number of sample data and sufficient climatic data were selected for this study to ensure the accuracy and generalizability of the results. This work can help us to understand the reasons for the differences in the quality of *Z. armatum* in different regions and provide a reference for *Z. armatum* in cultivation and planting site selection.

## 2. Results

### 2.1. Quantitative Analysis of Volatile Components of Z. armatum Peels

**Figure 1:**
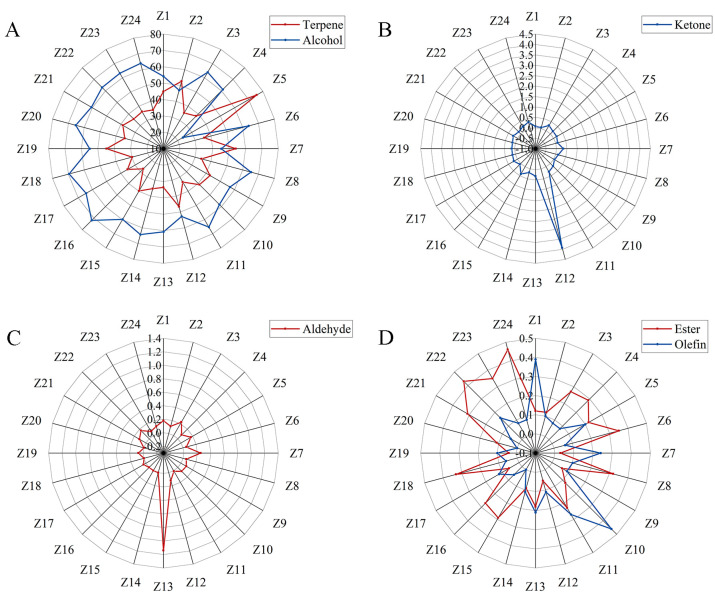
Distribution of volatile matter content composition of Chinese prickly ash peels from different producing areas. (**A**) distribution of terpene and alcohol components; (**B**) distribution of ketone component; (**C**) distribution of aldehyde component; (**D**) distribution of ester and olefin component.

In this experiment, 24 areas of *Z. armatum* were selected for the study, hereafter referred to as Z1–24. Specific origin information is shown in Table S1. The essential oils were analyzed for their compositions and relative contents using GC/MS. A total of 145 volatile components were detected in 24 samples from different origins (Table S2). The highest number of terpenes was 68, followed by alcohols (27), aldehydes (13), lipids (16), ketones (8), olefins (9), acids (2), and others (1). Figure 1 shows the variation in essential oil content of different major groups in different regions. From Figure 1A, it can be seen that the relative content of alcohols was the highest in most *Z. armatum* samples, except for Z5 and Z7, which were all higher than 50%, especially in Z16, which was as high as 71.88%. The relative content of olefins was also high, except for Z16, which was higher than 30%, and Z5, which was up to 75.6%. The ketone was little in all the other origins except for Z12, where the relative content was up to 3.92% (Figure 1B). Aldehyde had a relative content of less than 0.3% in all the other origins and 1.14% in Z13 (Figure 1C). Ester varied greatly among *Z. armatum* samples, but none of them was more than 0.3% (Figure 1D). Olefin did not exceed 0.2% in most of the *Z. armatum* samples, with a maximum of 0.46% in Z10 (Figure 1D). Acid was only detected in Z13, with 6-octadecenoic acid (0.1%) and n-hexadecanoic acid (0.05%). Heptadecyl oxirane was not in these categories and was detected only in Z10 (0.02%) and Z22 (0.03%) in very trace amounts. Thus, terpenes and alcohols should be the main active ingredients in the peels of *Z. armatum*.

### 2.2. Main Flavor Compounds of Z. armatum

The odor and quality of *Z. armatum* peels are mainly determined by the content and composition of essential oils [6]. Only 24 of all volatile compounds were queried for odor thresholds [21,22,23] (Table 1). Terpenes were the most abundant, with seven species, followed by alcohols (6), aldehydes (6), lipids (5), and acids (1). The highest content was linalool, with an average relative content of 55.65%, followed by d-limonene, with an average relative content of 39.97%. All other substances with odor characteristics had less average relative content, such as caryophyllene (1.28%), terpinen-4-01 (0.93%), and terpinolene (0.56%). The highest relative content of linalool in *Z. armatum* and the lower odor threshold (7.4 μg/kg) contributed the most to the flavor, thus defining ROAV_stan_ = 100 for linalool. The larger the ROAV value, the greater the contribution to the overall flavor of the sample. Compounds with ROAV values ≥ 1 were the key flavor compounds of the samples; compounds with ROAV values between 0.1 and 1 were important modifiers of the overall flavor of the samples [24]. Based on Table 1, we identified nine volatile components as the main flavor compounds of *Z. armatum*. Among them, linalool, decanal, and d-limonene had ROAV values greater than 1 and were the key flavor compounds of *Z. armatum*. Linalool presented a sweet, floral, kale, and lavender odor; decanal had a fresh, greasy, and fruity aroma; and d-limonene smelled like fresh citrus and mint. Therefore, *Z. armatum* is mainly characterized by spicy, floral, oily, and fruity aromas. In addition to this, the other subject flavor compounds are caryophyllene, α-copaene, dodecanal, methyl isocaproate, geranyl acetate, and geranyl isobutyrate.

### 2.3. HCA and PCA Analysis of the Main Flavor Compounds

**Figure 2:**
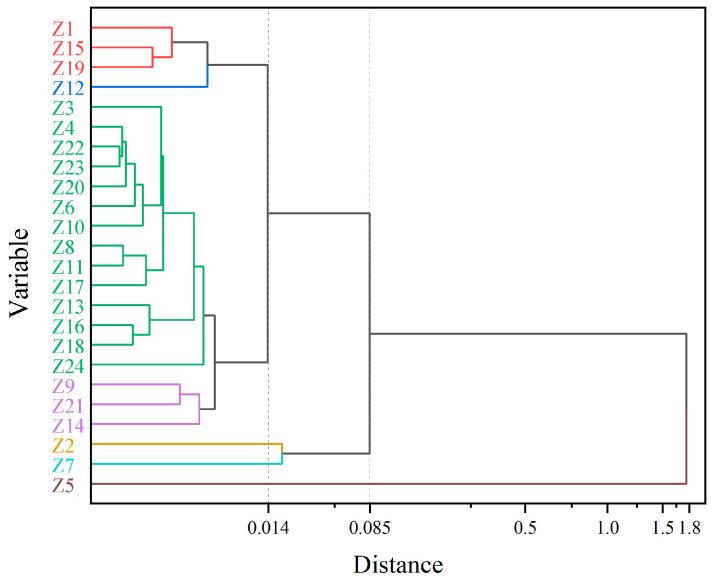
Dendrograms resulting from hierarchical clustering analysis of Chinese prickly ash from different locations. Samples that are divided into the same group are indicated by the same color.

**Figure 3:**
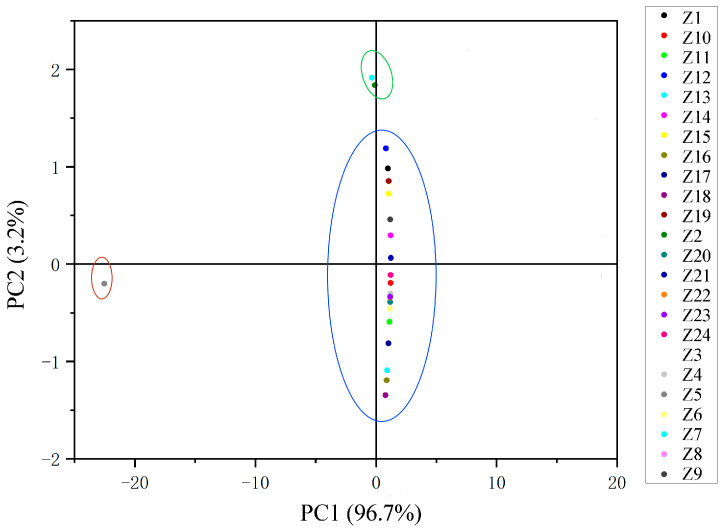
Two-dimensional scores plot of the PCA of 24 samples, and each color represents one sample.

The main flavor compounds were selected among all essential oils for hierarchical cluster analysis (HCA) and principal component analysis (PCA). Pearson’s correlation between the relative contents of the nine main flavor compounds screened was first calculated, then normalized by the Z-value method, and finally, a cluster diagram was obtained (Figure 2). The relative amounts of these nine main flavor compounds in each sample are shown in Table S3. When the distance coefficient was equal to 0.085, the samples were divided into three groups. Among them, Z5 was divided into a separate group, Z2 and Z7 were divided into one group, and the remaining samples were gathered into one group. The essential oil content of Z5 was significantly different from other *Z. armatum* samples. The relative content of linalool was much lower than that of the other samples, only 16.81%, and the relative content of d-limonene was much higher than that of the other samples, reaching 29.38%. The relative content of the other essential oils of Z5 was also the same, either being the highest or the lowest of all the samples, presenting the exact opposite variation from that of the other samples. The relative amounts of linalool, d-limonene, caryophyllene, and decanal were very similar in Z2 and Z7, and none of the four compounds, α-copaene, dodecanal, geranyl acetate, and geranyl isobutyrate, were detected. When the distance coefficient was 0.014, all samples were divided into five groups. In the principal component analysis, PC1 was 96.7% and PC2 was 3.2%. All samples were also categorized into three groups, which is consistent with the results of HCA (Figure 3).

### 2.4. Correlation Analysis of Main Flavor Compounds with Climatic Factors

**Figure 4:**
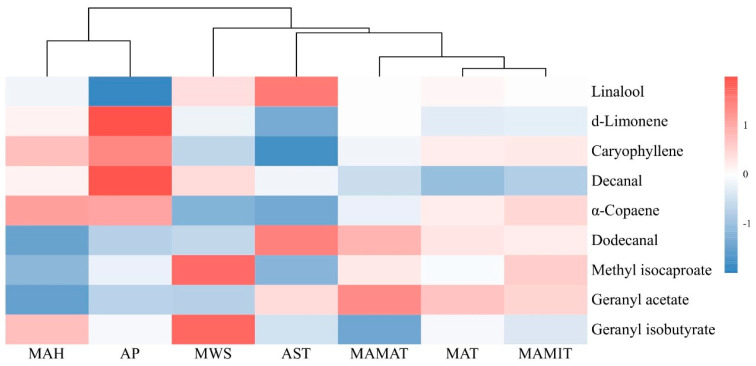
Results of correlation analysis between volatile components and climatic factors. The mean annual temperature (MAT), mean annual minimum temperature (MAMIT), mean annual maximum temperature (MAMAT), mean annual humidity (MAH), annual precipitation (AP), annual sunshine time (AST), and mean annual wind speed (MWS).

Correlations between the analysis of subject flavor compounds and climatic factors were selected to analyze the relationship between climate and flavor formation in *Z. armatum*. The results showed that different essential oils showed different correlations with climatic factors (Figure 4). The correlation coefficient is listed in Table S4. All climate factors were clustered into three groups: MAH and AP were clustered together, MWS and AST were clustered together, and the remaining MAMAT, MAT, and MAMIT were clustered together. MAMAT, MAT, and MAMIT were all related to temperature, and MAH and AP were all related to precipitation, so it was reasonable to be clustered in the same group. Linalool was positively correlated with MAMAT, MAMIT, MWS, and AST and negatively correlated with MAT, MAH, and AP. Two of the climate factors positively correlated with linalool were temperature-related, and most of the negatively correlated climate factors were precipitation-related. The essential oil of d-limonene was the opposite of linalool. The essential oil of d-limonene was positively correlated with MAH and AP, which were correlated with precipitation and negatively correlated with all other climate factors. Compounds are categorized according to three groups: the essential oils d-limonene, caryophyllene, decanal, and α-copaene were positively correlated with MAH and AP, and dodecanal, methyl isocaproate, and geranyl acetate were negatively correlated with MAH and AP. MWS and AST were positively correlated with linalool and negatively correlated with d-limonene, caryophyllene, and α-copaen. MAMAT, MAT, and MAMIT were positively correlated with caryophyllene, α-copaene, and geranyl acetate and negatively correlated with d-limonene, decanal, and geranyl isobutyrate were negatively correlated. In general, most of the positive and negative relationships between the same essential oils and temperature and precipitation-related climatic factors were consistent. Thus, the essential oil of Chinese prickly ash peels is mainly influenced by two climatic factors: temperature and precipitation.

### 2.5. Path Analysis

To further determine the relationship between climate factors and essential oil, another path analysis was performed (Table 2). Firstly, a stepwise regression analysis was performed between climate factors and essential oils, and *p* < 0.05 was chosen for the next step of the analysis to determine their dominant climate factors. Ultimately, only five essential oils showed a strong linear relationship with climate factors. Linalool, d-limonene, (−)-β-elemene, and β-ocimene dominated the climate factors as AP. β-thujene was mainly influenced by MAMIT. d Germacrene D and (−)-α-selinene dominated the climate factors AP and MWS. Caryophyllene is dominated by MAMAT and MWS. The dominant climate factor for (*E*)-nerolidol was MAMAT. z,z,z-1,5,9,9-tetramethyl-1,4,7,-Cycloundecatriene was dominated by MWS in addition to RD as its dominant climate factor.

As shown by the results of the path analysis, AP had a large direct positive effect on d-limonene (0.741) and the lowest correlation coefficient (0.000). AP had a relatively small direct positive effect on decanal (0.573) with a correlation coefficient of 0.10. AP had the smallest direct negative effect (−0.678) on linalool with a correlation coefficient of 0.001. MAMAT had a direct negative effect on caryophyllene when it was used as a direct influence factor (−0.607), with a correlation coefficient of 0.001, at which time MWS, as an indirect influence factor, had an indirect positive effect (0.358). MWS showed a direct positive effect when it directly influenced caryophyllene (0.813), and MAMAT showed an indirect positive effect (0.358). Thus, essential oils were mainly affected by precipitation.

## 3. Discussion

Essential oils are compounds with aromatic characteristics that have many biological activities [8]. Linalool has a moderating effect on pain [25]. Decanal has bacteriostatic bioactivity [26]. The d-limonene has biological activities such as hypoglycemic and free radical scavenging [27]. *Z. armatum* peels are the source of flavor for *Z. armatum*, so we analyzed the essential oil composition and content of the peels of *Z. armatum* [28]. The effects of different habitats on the essential oil content varied greatly. Ghasemi et al. [29] found that the content of essential oils of *Ferulago angulata* collected from different natural habitats varied according to chemotype, environmental conditions, and geographical origin. In this paper, 24 samples of *Z. armatum* were selected from *Z. armatum* habitats in Southwest China, and GC-MS analysis revealed the largest number of terpenes and the largest relative content of alcohols. The same conclusion was reached in the study of Hanyuan *Z. bungeanum* by Zhao et al. [30]. Terpenes, alcohols, ketones, and alcohols were found to be the major essential oil constituents of *Z. armatum* in a previous study, which is in agreement with our findings [30,31]. Linalool was also found to be the major essential oil constituent of *Z. armatum* in all the samples in this study, and its content was much higher than that of d-limonene. In the analysis of the essential oil content of *Z. aramatum* from Delhi, 57.0% of linalool and 19.8% of d-limonene were found by Tiwary et al. [32]. This is in agreement with our findings. In this work, a total of nine subject flavor compounds were identified in 24 batches of *Z. armatum* samples, of which linalool was defined as ROAV_stan_ = 100. Liu et al. identified 32 odor-active compounds from *Zanthoxylum armatum* DC collected from Xichang City, among which linalool had the highest flavor dilution (FD) factors, concentration, and odor activity value (OAV) [33].

The HCA results show that there is little connection between the essential oils of *Z. armatum* and regions. For example, Z21, Z22, Z23, and Z24 were all picked from Bazhong; Z17, Z18, and Z19 were all from Guang’an; and Z8, Z10, and Z11 were all from Zigong. None of them were well clustered together. This may be related to the instability of essential oils [34]. Zhuo et al. [35] found in their analysis of 24 batches of *Z. armatum* peels that two batches of samples were both from Bazhong City, but both PCA and HCA classified them into two different groups. It is hypothesized that processing methods, climatic conditions, cultivars, and other factors in different origins may lead to large differences in the volatile components [36,37,38]. Also, when harvested and transported inappropriately, the oil droplets on the surface of the essential oils can break up, resulting in lower levels [6]. Z5 is from Hongya County, Meishan City, and the climatic factors and geographic conditions of its region are not particularly different from other *Z. armatum*-producing areas, and it is hypothesized that the large differences may be due to improper harvesting or transportation.

Essential oils are secondary metabolites of plants, and the accumulation of secondary metabolites is closely related to environmental changes, so choosing the right cultivation conditions is crucial [15]. In the effect of climatic factors on *Z. armatum*, we found that most of the nine essential oils screened were affected by temperature and precipitation. Dougnon et al. [39], in their study of essential oils of leaves of *Melia azedarach* L., found a positive correlation between the amount of precipitation and temperature and the content of the essential oils and categorized these oils into four groups based on the correlation properties. Acimovic et al. [40] regression modeled Lavandin essential oils based on temperature and precipitation data and also found a correlation between them. Guelsoy et al. [41], in exploring the effect of environmental factors on the essential oil of wild *Pistacia terebinthus* L. fruits, found that precipitation was decisive for d-limonene. Correlation analysis also revealed a negative correlation between d-limonene, caryophyllene, decanal, α-copaene, and geranyl acetate and temperature.

## 4. Materials and Methods

### 4.1. Plant Materials

**Figure 5:**
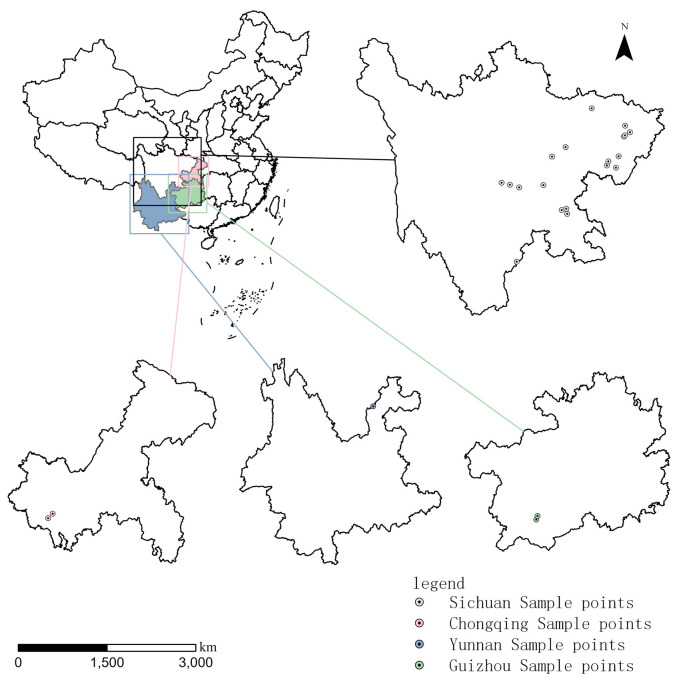
Map of collection sample sites of *Zanthoxylum armatum* DC.

*Z. armatum* samples were selected from 24 main prickly ash-producing areas through the local Forestry Bureau or prickly ash planting companies (Figure 5). The provinces of Sichuan, Yunnan, Guizhou, and Chongqing are the main production areas of *Z. armatum* [6]. In this experiment, the fruits of *Z. armatum* with better appearance and quality were selected for harvesting in May–July 2022. The collected *Z. armatum* fruits were dried and deseeded to obtain dry pericarp (moisture content less than 10.5%), with a sampling volume of 5–10 kg. The dry pericarp was crushed, passed through a 60-mesh sieve, and stored in a refrigerator at −20 °C for backup.

### 4.2. Essential Oil Extraction

*Z. armatum* was pulverized after removing impurities, 40 g of powdered sample was transferred into a 500 mL round-bottomed flask, 400 mL of deionized water was added, connected with a condenser tube, the flask was heated, and the distillation rate was adjusted to 2 mL/min~3 mL/min. Distillation was carried out for 4 h [41]. The yellow liquid obtained was stored in the refrigerator at (−20 ± 1) °C after removing the water with anhydrous sodium sulfate for backup. Three repeated tests were conducted for all experiments.

### 4.3. GC/MS Conditions

The essential oil was diluted 40 times with methanol, passed through a 0.22 µm filter membrane, and 1 mL was injected into an auto-sampling vial with an injection volume of 1 µL. The chromatographic column was an HP-5MS flexible quartz capillary column (30 m × 0.25 mm, 0.25 μm). The warming procedure was held at a column temperature of 50 °C as the starting temperature for 1 min, and then the warming was started. The temperature was first increased to 75 °C at a rate of 1 °C/min (1 min), then increased to 120 °C at a rate of 6 °C/min (1 min), followed by 135 °C at a rate of 1 °C/min and held for 1 min, and finally, increased to 200 °C at a rate of 15 °C/min and held for 5 min [42]. Helium was used as the carrier gas at a flow rate of 1.0 mL/min, with a spacer purge flow rate of 3 mL/min, a pressure of 7.6522 psi, and a temperature of 250 °C at the inlet. The ion source was an electron impact ionization source (EI), the ion source temperature was 230 °C, the quadrupole was 150 °C (maximum value 200 °C), the electron energy was 70 eV, the interface temperature was 280 °C, and the mass scanning range was 50~550 amu.

### 4.4. Climate Factor Data

This study analyzed the factors affecting the essential oil of *Z. armatum* about climatic factors. The mean annual temperature (MAT), mean annual minimum temperature (MAMIT), mean annual maximum temperature (MAMAT), mean annual humidity (MAH), annual precipitation (AP), annual sunshine time (AST), and mean annual wind speed (MWS) in the sampling area were provided by the Sichuan Meteorological Bureau and China Meteorological Data (https://data.cma.cn/, accessed on 20 December 2023) (Table S5).

### 4.5. Statistical Analysis

The relative odor activity (ROAV) method was used to evaluate the contribution of volatile components to the flavor of *Z. armatum* [43]. ROAV*_stan_* = 100 was defined as the component that contributed the most to the flavor of the sample, and for the other components, it was calculated according to the following formula.

$${ROAV}_{i}=100\times \frac{C_{i}}{C_{stan}}\times \frac{T_{stan}}{T_{i}}$$

Among them, *C_i_* and *C_stan_* are the relative contents (%) of each volatile component and the component that contributes most to the flavor in the samples, respectively, and *T_i_* and *T_stan_* are the odor thresholds (μg/kg) of each volatile component and the component that contributes most to the flavor in the samples, respectively.

Calculations for hierarchical cluster analysis, correlation analysis, regression analysis, and path analysis were performed using IBM SPSS Statistics 27 (International Business Machines Corporation, New York, NY, USA). Hierarchical cluster analysis plots, principal component analysis plots, and correlation analysis plots were generated by Origin 2022 (OriginLab, Northampton, MA, USA).

## 5. Conclusions

Environmental factors are important factors affecting the accumulation of secondary metabolites; therefore, this paper analyzed the relationship between the essential oils of *Z. armatum* and climatic factors in 24 regions. The GC/MS results showed that linalool and d-limonene were prevalent in 24 batches of *Z. armatum* samples. ROAV results showed that linalool, decanal, and d-limonene were the key main flavor compounds of peppercorns, which gave *Z. armatum* to present pungent, floral, oily, and fruity odors. Both linalool and d-limonene were dominated by the climatic factor of precipitation in the path analysis, with direct correlation effects of −0.678 and 0.741, respectively. However, in the correlation analysis, temperature was found to be positively correlated with linalool and negatively correlated with d-limonene. This study provides a reference for the identification of the flavor and quality of *Z. armatum*, as well as climatic factor references for the selection of *Z. armatum* in the production area. But there are more factors than just climate that affect essential oils, and altitude, soil, and many other factors can be considered in subsequent studies.

## Figures and Tables

**Table 1:** The ROAV value of volatile components in *Z. armatum*.

Number	Compound Name	ROAV	Odor Characteristics [21,22,23]
1	Linalool	100.00	Sweet, floral scent reminiscent of kale and lavender
2	d-Limonene	1.11	Fresh citrus and mint
3	Caryophyllene	0.27	Pale lilac-like scent
4	Terpinolene	0.04	Lemon scent
5	L-α-Terpineol	0.02	Clove flavor
6	(*Z*)-3,7-dimethyl-2,6-Octadien-1-ol	0.01	Nearly sweet aroma of fresh roses, slightly lemony
7	α-Phellandrene	<0.01	Has a weedy, aniseed flavor
8	Decanal	9.71	Fresh grease aroma, fruity
9	α-Copaene	0.19	Pine and turpentine aroma
10	Undecanal	0.09	Rose, floral, fruity, and sweet orange aroma.
11	Tetradecanal	0.01	Oily and iris-like aroma.
12	α-Pinene	<0.01	Terpene-like and minty
13	Copaene	0.07	Pine and turpentine aroma
14	α-Terpineol	0.03	Strong floral aroma, typical clove flavor
15	Geraniol	0.04	Has a mild, sweet rose scent
16	(*R*)-3,7-Dimethyl-6-octenal	0.02	Strong, fresh herbal citrus flavor
17	Citronellal	0.03	Citrus flavor
18	Dodecanal	0.16	Soap, wax, aldehydes, citrus, and violet flowers
19	Methyl isocaproate	0.17	Sweet fruity, etheric aroma
20	Bornyl acetate	0.01	Woody, camphoraceous, minty, cypress, spicy and soapy aroma
21	L-Bornyl acetate	<0.01	Aroma of forest freshness
22	Geranyl acetate	0.28	Aroma of rose, bergamot, and lavender
23	Geranyl isobutyrate	0.14	Pale rose aroma and sweet apricot aroma
24	n-Hexadecanoic	<0.01	Slightly fatty, waxy aroma

**Table 2:** Path analysis between climate factors and volatile component of Chinese prickly ash peels.

Item	Factors	Correlation Coefficients	Direct Path Coefficients	Indirect Path Coefficient		Significance Level
Linalool	AP	−0.678	−0.678			0.001
d-Limonene	AP	0.741	0.741			0.000
				MWS	MAMAT	
Caryophyllene	MAMAT	−0.316	−0.607	0.358		0.001
	MWS	0.596	0.813		0.358	0.000
Decanal	AP	0.573	0.573			0.010
Geranyl acetate	MAH	−0.623	−0.623			0.004

## Data Availability

The data supporting the results are available in a public repository at: Qianqian Qian (2023). GC-MS data of essential oils of *Zanthoxylum armatum* DC. from different production areas. figshare. Dataset. https://doi.org/10.6084/m9.figshare.24523540.v1.

## Supplementary Materials

The following supporting information can be downloaded at: https://www.mdpi.com/article/10.3390/molecules29061343/s1, Table S1: Sample source information of *Zanthoxylum armatum* DC; Table S2: Volatile components in *Zanthoxylum armatum* DC. adetected by GC/MS; Table S3: Relative amounts of nine major flavor compounds in each sample; Table S4: Results of person correlation analysis between volatile components and climatic factors; Table S5: Climate data averaged over the last 5 years (2017–2022) for Chinese prickly ash producing areas in China.
