# Supplementary material for: Chemical Composition Variation in Essential Oil and Their Correlation with Climate Factors in Chinese Prickly Ash Peels (Zanthoxylum armatum DC.) from Different Habitats
Source: Molecules. 2024 Mar 18;29(6):1343. doi: 10.3390/molecules29061343 (PMC10974008; doi:10.3390/molecules29061343)
Supplement: Supplementary file 1 [file molecules-29-01343-s001.zip › Table S1.pdf]

Table S1 Sample source information of *Zanthoxylum armatum* DC

Sample Code	Place of origin (province, city/state, county/district)	Longitude (°)	Latitude (°)	Altitude (m)	Age of tree (year)
Z1	Sichuan, Ya'an, Tianquan	102.9	30.0	726	4
Z2	Sichuan, Ya'an, Yucheng District	103.1	30.0	610	4
Z3	Yunnan, Zhaotong, Yanshan	103.2	27.5	2015	9
Z4	Sichuan, Liangshan, Jinyang	103.3	27.7	2050	7
Z5	Sichuan, Meishan, Hongya	103.3	29.9	933	5
Z6	Sichuan, Meishan, Renshou	104.2	30.0	402	5
Z7	Sichuan, Chengdu, Jintang	104.5	30.8	448	4
Z8	Sichuan, Zigong, Yantan District	104.8	29.2	385	4
Z9	Sichuan, Mianyang, Santai	104.9	31.3	434	4
Z10	Sichuan, Zigong, Yantan District	105.0	29.3	322	4
Z11	Sichuan, Zigong, Fushun	105.2	29.0	281	4
Z12	Sichuan, Guangyuan, Zhaohua District	105.6	32.2	660	4
Z13	Guizhou, Qianxinan, Zhenfeng	105.6	25.6	628	7
Z14	Guizhou, Qianxinan, Zhenfeng	105.7	25.7	614	7
Z15	Chongqing, Jiangjin	106.2	29.1	311	6
Z16	Chongqing, Jiangjin	106.3	29.2	315	6
Z17	Sichuan, Guang'an, Yuechi	106.5	30.6	436	4
Z18	Sichuan, Guang'an, Yuechi	106.5	30.7	389	4
Z19	Sichuan, Guang'an, Qianfeng District	106.6	30.5	683	4
Z20	Sichuan, Dazhou, Quxian	106.8	30.9	338	5
Z21	Sichuan, Bazhong, Pingchang	107.0	31.4	618	5
Z22	Sichuan, Bazhong, Pingchang	107.1	31.4	385	5
Z23	Sichuan, Bazhong, Pingchang	107.3	31.7	663	5
Z24	Sichuan, Bazhong, Pingchang	107.4	31.8	432	5

Note: *Z. armatum* samples are live seedlings.
