# Supplementary material for: Chemical Composition Variation in Essential Oil and Their Correlation with Climate Factors in Chinese Prickly Ash Peels (Zanthoxylum armatum DC.) from Different Habitats
Source: Molecules. 2024 Mar 18;29(6):1343. doi: 10.3390/molecules29061343 (PMC10974008; doi:10.3390/molecules29061343)
Supplement: Supplementary file 1 [file molecules-29-01343-s001.zip › Table S2.pdf]

Table S2 Volatile components in *Zanthoxylum armatum* DC. adetected by GC/MS

Number	Compound name	Molecular formula	Retention index (RI)	Odor threshold µg/kg	Odor characteristics	Relative content/%																							
						Z1	Z2	Z3	Z4	Z5	Z6	Z7	Z8	Z9	Z10	Z11	Z12	Z13	Z14	Z15	Z16	Z17	Z18	Z19	Z20	Z21	Z22	Z23	Z24
	Terpene																												
	3-Thujene	$C_{10}H_{16}$	852			—	—	—	0.63	0.67	—	0.59	0.41	0.56	—	0.34	0.49	0.48	0.58	—	—	0.24	—	0.42	0.33	0.36	0.27	0.41	—
2	β-Thujene	$C_{10}H_{16}$	852			10.28	7.48	0.72	7.52	—	0.48	7.25	4.77	8.61	0.39	—	6.08	9.39	5.14	—	—	2.14	0.33	4.74	4.72	5.31	8.53	3.47	0.32
3	Cyclofeuchene	$C_{10}H_{16}$	854			—	—	—	—	—	—	—	—	—	0.32	0.31	—	—	—	—	—	—	—	—	—	—	—	—	—
4	(1S)-(-)-alpha-Pinene	$C_{10}H_{16}$	854			—	—	—	—	—	0.55	0.80	—	—	—	—	0.44	—	—	—	—	—	—	—	—	—	0.49	—	—
5	α-Pinene	$C_{10}H_{16}$	854	2100	Terpene-like and minty	—	—	—	—	—	—	—	—	—	—	—	—	—	—	—	—	0.18	—	—	—	—	—	—	—
6	(1r)-(+)- alpha-pinene	$C_{10}H_{16}$	855			—	0.53	0.78	0.66	—	—	—	0.45	0.36	—	—	—	0.69	—	0.53	0.25	—	0.27	0.50	0.33	0.58	—	0.40	0.32
7	1,7,7- Trimethyl-tricyclo [2.2.1.0(2,6)] heptane	$C_{10}H_{16}$	855			0.34	—	—	—	—	—	—	—	—	—	—	—	—	—	—	—	—	—	—	—	—	—	—	—
8	Sabenene	$C_{10}H_{16}$	880			—	5.00	—	4.96	11.17	3.50	—	—	—	—	6.76	3.57	—	3.39	—	2.71	3.19	3.20	4.61	3.20	—	—	4.94	3.78

9	β-Humulene	$C_{15}H_{24}$	891			0.97	3.13	2.29	2.74	5.94	2.82	4.75	2.65	3.27	3.02	2.60	4.14	—	0.84	3.40	2.65	2.25	2.22	3.40	2.07	3.10	2.83	2.88	2.25
10	β-Phellandrene	$C_{10}H_{16}$	892			—	1.66	9.35	—	—	5.42	6.83	3.02	—	7.62	—	—	4.60	—	7.39	1.78	0.66	1.94	—	—	3.77	—	—	3.84
11	α-Phellandrene	$C_{10}H_{16}$	1001	700	With weed herbaceous, aniseed flavor	0.20	0.22	0.22	0.24	0.43	0.24	—	0.27	0.25	0.16	0.32	0.19	0.21	0.08	0.32	—	—	—	0.30	0.26	0.30	0.33	0.29	0.40
12	2-Carene	$C_{10}H_{16}$	1004			—	—	—	0.12	1.16	0.61	—	—	—	—	—	—	0.68	0.33	0.23	0.19	—	—	—	—	—	0.12	—	—
13	(+)-4-Carene	$C_{10}H_{16}$	1004			—	—	—	—	0.23	—	—	0.12	—	—	—	—	—	—	—	—	—	—	0.44	—	0.46	—	—	0.09
14	α-Terpinen	$C_{10}H_{16}$	1004			0.65	0.77	0.29	0.88	—	—	0.06	—	0.60	0.53	0.91	0.79	—	—	—	—	—	0.42	0.68	0.63	0.10	—	0.71	—
15	d-Limonene	$C_{10}H_{16}$	1013	200	Fresh citrus Orange, Mint Flavors	19.78	22.97	15.29	14.71	29.38	14.46	22.85	13.95	17.27	14.91	14.09	20.67	11.23	19.17	18.81	10.77	13.34	11.37	19.11	14.91	16.11	15.05	15.12	16.29
16	trans-β-Ocimene	$C_{10}H_{16}$	1018			0.21	0.24	0.26	0.30	0.93	0.22	0.17	0.23	0.17	0.17	0.20	0.25	0.21	0.18	0.33	0.41	0.26	0.23	0.16	0.22	0.40	0.26	0.26	0.28
17	β-Ocimene	$C_{10}H_{16}$	1020			0.14	1.34	—	—	2.13	0.73	1.17	0.78	—	—	0.86	1.08	0.62	0.62	0.81	—	—	0.69	1.04	0.70	0.99	0.12	—	—
18	(Z)-β-ocimene	$C_{10}H_{16}$	1023			0.95	—	0.73	0.93	—	—	—	—	0.91	0.72	0.03	—	—	—	—	0.82	0.78	—	—	—	0.08	0.83	0.83	1.07
19	γ-Terpinene	$C_{10}H_{16}$	1027			0.9	1.0	0.8	1.3	1.5	0.9	1.1	1.0	0.9	0.8	1.1	1.1	1.1	1.0	0.7	0.6	0.6	0.8	0.8	1.0	1.1	0.9	0.9	0.8

						0	5	6	7	8	1	8	6	6	1	6	2	9	2	9	2	3	8	3	6	1	4	0	4
20	Terpinolene	$C_{10}H_{16}$	1042	200	lemony	0.4	0.5	0.3	0.4	0.8	0.3	1.2	1.1	0.5	0.4	0.3	0.5	0.4	0.3	0.3	0.2	0.6	0.2	—	0.3	0.6	0.9	0.4	1.0
					Taste	7	2	5	9	3	4	3	1	0	1	9	3	6	8	3	4	6	7		6	9	2	4	2
21	α-Pyronene	$C_{10}H_{16}$	1068			—	—	—	—	—	—	—	0.0	—	—	—	—	—	—	—	—	—	—	—	—	—	—	—	—
													9																
22	Alloocimene	$C_{10}H_{16}$	1068			0.0	0.0	0.0	0.1	0.1	0.0	0.0	—	0.0	0.0	0.0	0.0	—	—	0.1	0.1	0.1	0.0	0.0	—	0.1	0.0	—	0.1
						7	7	9	0	7	9	5		8	6	7	8			7	8	0	9	7		1	9		0
23	(±)-m-Mentha-	$C_{10}H_{16}$	1093			—	—	—	—	—	0.0	0.2	—	—	0.0	0.8	0.4	—	—	—	0.8	—	—	—	0.7	0.7	0.7	0.7	—
	1,8-diene										6	9			4	0	5				3				9	9	6	9	
24	Ocimene	$C_{10}H_{16}$	1224			—	—	—	—	—	—	—	0.0	0.0	—	—	0.1	—	—	0.3	—	—	—	—	—	—	—	—	—
													4	7			2			1									
25	(+)-3-Carene	$C_{10}H_{16}$	1224			—	—	—	0.0	—	—	—	—	—	—	—	—	—	—	—	—	—	—	—	—	—	—	—	—
									5																				
26	3-Carene	$C_{10}H_{16}$	1224			—	—	—	—	—	—	—	—	—	—	—	—	—	—	—	—	—	—	—	—	—	—	0.0	—
																												4	
27	(-)-γ-Elemene	$C_{15}H_{24}$	1263			—	—	0.1	0.1	—	—	—	—	—	—	0.1	—	0.1	0.1	—	—	—	—	—	—	—	—	—	—
								7	2							8		6	4										
28	2,5,6-Trimethyl-	$C_{10}H_{16}$	1263			—	—	—	—	—	—	—	—	—	0.3	—	—	—	—	—	—	—	—	—	—	—	—	—	—
	1,3,6-heptatriene														2														
29	1,5,5-Trimethyl-	$C_{10}H_{16}$	1264			—	—	—	—	—	—	—	—	0.2	—	—	—	—	—	0.1	—	0.3	—	—	—	0.1	—	—	0.1
	6-methylene-													6						8		4				4			5
	cyclohexene																												
30	α-Cubebene	$C_{15}H_{24}$	1270			—	—	—	—	—	—	—	—	0.0	—	—	0.0	—	—	—	—	—	—	—	—	—	0.0	—	0.0
														3			3										3		3
31	α-Copaene	$C_{15}H_{24}$	1283	6	With pine	0.0	—	—	—	0.0	—	—	0.0	0.0	—	—	—	—	—	—	—	—	0.2	—	—	—	—	—	—
					Wood,	5				8			3	3									4						

					turpentine oil aroma																								
32	Copaene	$C_{15}H_{24}$	1284	6	With pine Wood, turpentine oil aroma	—	0.03	—	—	—	—	0.03	—	—	0.04	0.03	—	—	—	—	—	—	0.03	0.03	—	0.03	—	—	—
33	β-Elemen	$C_{15}H_{24}$	1289			0.25	2.14	0.15	0.05	0.43	—	0.13	—	1.72	3.34	—	0.14	0.13	0.10	0.75	—	0.21	1.27	0.18	—	—	—	—	—
34	(-)-β-Elemene	$C_{15}H_{24}$	1294			2.50	—	—	—	4.95	0.73	1.67	0.42	—	—	0.22	1.36	—	—	—	0.46	2.89	—	1.90	0.43	0.28	0.28	0.36	0.29
35	Caryophyllene	$C_{15}H_{24}$	1406	64	Pale lilac Fragrant	1.56	1.31	0.82	0.71	3.43	1.05	1.24	1.05	1.70	1.66	1.11	1.16	0.94	0.86	1.36	1.16	1.80	1.32	1.56	1.07	0.86	0.97	1.05	0.92
36	(-)-β-cubebene	$C_{15}H_{24}$	1410			0.13	0.10	—	0.05	—	—	0.09	0.09	0.12	0.21	—	—	—	—	0.09	0.09	0.14	0.12	—	0.08	0.08	0.08	0.09	—
37	γ-Elemene	$C_{15}H_{24}$	1412			0.10	0.08	—	0.24	0.20	—	0.06	—	—	0.11	—	0.06	—	—	—	—	0.11	0.08	0.08	—	0.45	—	0.51	—
38	α-Guaiene	$C_{15}H_{24}$	1414			0.04	—	—	—	—	—	—	—	0.05	—	—	—	—	—	—	—	—	—	—	—	—	—	—	—
39	γ-Muurolene	$C_{15}H_{24}$	1416			0.33	0.05	—	0.11	0.54	—	—	0.13	—	—	0.04	—	—	—	0.15	—	0.16	—	—	—	—	0.33	0.12	0.14
40	Humulene	$C_{15}H_{24}$	1420			—	—	—	—	—	—	—	—	—	—	—	—	—	0.41	—	0.75	—	—	—	0.64	0.54	—	—	—
41	Z, Z, Z-1,5,9,9-tetramethyl-1,4,7,-	$C_{15}H_{24}$	1420			0.52	0.42	0.43	0.33	1.19	0.55	0.37	0.65	0.67	0.55	0.60	0.39	0.36	—	0.83	—	0.61	0.66	0.59	—	—	0.52	0.56	0.53

	Cycloundecatriene																										
42	cis-β-Farnesene	$C_{15}H_{24}$	1423	—	—	0.03	—	—	0.07	—	0.09	0.06	—	0.06	—	—	—	0.07	0.08	—	0.08	0.05	—	—	0.06	0.06	0.06
43	(E)-β-Famesene	$C_{15}H_{24}$	1423	—	—	—	—	—	—	—	—	—	—	—	—	—	—	—	—	—	—	—	0.09	0.08	—	—	—
44	β-Farnesene	$C_{15}H_{24}$	1423	—	—	—	—	—	—	—	—	—	0.04	—	—	—	—	—	—	—	—	—	—	—	—	—	—
45	(+)-epi-Bicyclosesquiphellandree	$C_{15}H_{24}$	1425	0.07	—	0.15	—	0.14	0.26	0.04	0.04	0.19	—	0.33	0.14	—	—	0.19	0.30	0.10	—	0.04	0.19	—	0.21	0.13	0.24
46	8-Isopropyl-5-methyl-2-methylene-1,2,3,4,4a,5,6, 7-octahydronaphthalene	$C_{15}H_{24}$	1425	—	0.05	0.08	—	—	—	0.04	0.23	—	0.15	—	0.05	0.08	0.07	—	—	—	0.10	—	—	0.20	—	—	—
47	D Germacrene D	$C_{15}H_{24}$	1434	2.01	1.59	1.44	0.83	4.82	1.26	1.52	1.26	1.90	2.09	1.40	1.61	1.25	1.22	1.42	1.69	2.14	1.68	1.98	1.16	1.17	1.29	1.30	1.19
48	(+)-β-Selinene	$C_{15}H_{24}$	1436	0.56	0.46	0.05	—	1.15	0.22	0.32	0.18	0.42	0.61	0.12	0.29	—	0.14	0.26	0.24	0.55	0.33	0.46	0.22	0.14	0.15	0.14	0.14
49	(-)-α-Selinene	$C_{15}H_{24}$	1440	1.39	1.10	—	—	3.08	0.66	0.94	—	1.14	1.56	—	0.90	—	—	—	0.68	1.45	0.96	1.15	0.57	—	—	—	—
50	(+)-Ledene	$C_{15}H_{24}$	1440	—	—	—	—	—	—	—	0.55	—	—	—	—	—	—	—	—	—	—	—	—	—	—	—	—

51	Bicyclogermacrene	$C_{15}H_{24}$	1441	—	—	0.42	—	—	—	—	—	—	—	—	—	0.37	0.47	—	—	—	—	—	—	—	—	—	—
52	α-Muurolene	$C_{15}H_{24}$	1444	—	—	—	—	—	—	—	—	0.07	—	—	—	—	—	—	—	—	0.12	—	—	—	—	—	—
53	α-Farnesene	$C_{15}H_{24}$	1448	—	—	—	—	—	—	—	—	—	—	—	—	—	—	—	—	—	0.04	—	—	0.04	—	—	—
54	(-)-α-Muurolene	$C_{15}H_{24}$	1450	0.16	—	0.11	—	0.52	0.36	0.11	—	0.46	0.42	0.34	0.23	0.21	—	0.15	—	—	0.18	—	—	—	0.03	—	0.14
55	(-)-γ-Cadinene	$C_{15}H_{24}$	1450	—	0.12	—	—	—	—	—	0.19	—	—	—	0.08	0.13	—	0.69	0.20	0.15	0.22	0.13	0.18	0.15	—	0.14	—
56	δ-Cadinene	$C_{15}H_{24}$	1455	0.16	—	0.11	0.08	0.46	0.15	0.11	0.19	0.20	0.18	0.18	0.14	0.13	0.09	0.16	0.20	0.20	0.30	0.14	—	0.15	0.14	0.15	—
57	(-)-β-Cadinene	$C_{15}H_{24}$	1455	—	—	—	—	—	—	—	—	—	—	—	—	—	—	—	—	—	—	—	0.21	—	—	—	0.13
58	cis-muurola-3,5-diene	$C_{15}H_{24}$	1455	—	0.13	—	—	—	—	—	—	—	—	—	—	—	—	—	—	—	—	—	—	—	—	—	—
59	α-Patchoulene	$C_{15}H_{24}$	1606	0.13	—	—	—	—	—	—	—	—	—	—	0.06	—	—	—	—	—	0.09	—	—	—	—	—	—
60	1,4-Dimethyl-3-(2-methyl-1-propene-1-yl)-4-vinyl-1-cycloheptene	$C_{15}H_{24}$	1606	—	—	—	—	—	—	—	—	—	0.19	—	—	—	—	—	—	—	—	0.11	—	—	—	—	—
61	γ-Gurjunene	$C_{15}H_{24}$	1607	—	—	—	—	—	—	—	—	0.10	—	—	—	—	—	—	—	—	—	—	—	—	—	—	—
62	γ-Maaliene	$C_{15}H_{24}$	1610	—	—	—	—	—	—	—	—	—	0.0	—	—	—	—	—	—	—	—	—	—	—	—	—	—

															4														
63	δ-selinene	$C_{15}H_{24}$	1622			—	—	—	—	—	—	—	—	—	—	0.07	—	0.10	—	—	—	—	—	—	—	—	—	—	—
64	(1.α.,4a.β.,8a.α.)-(.+/-.)-1,2,4a,5,8,8a-hexahydro-4,7-dimethyl-1-(1-methylethyl)naphthalene	$C_{15}H_{24}$	1622			—	—	—	—	—	—	—	—	—	0.06	—	—	—	—	—	—	—	—	—	—	—	—	—	—
65	β-Maaliene	$C_{15}H_{24}$	1638			—	—	0.06	—	—	—	—	—	0.04	—	—	—	—	—	—	0.08	—	—	—	—	0.04	0.05	—	—
66	β-Panasinsene	$C_{15}H_{24}$	1639			—	—	—	—	—	—	—	—	—	0.29	—	—	—	—	—	—	—	—	—	—	—	—	—	—
67	Valencen	$C_{15}H_{24}$	1638			—	—	—	—	—	—	0.13	—	—	—	—	—	—	—	—	—	—	—	—	—	—	—	—	—
68	Eremophilene(7CI)	$C_{15}H_{24}$	1639			—	0.11	—	—	—	—	—	—	—	—	—	—	—	—	—	—	—	—	—	—	—	—	—	—
	∑					44.93	52.69	35.23	38.21	75.60	35.75	54.02	34.02	42.77	41.03	33.51	46.61	33.63	35.23	39.72	27.40	35.27	29.73	44.68	34.40	38.60	35.66	36.09	34.55
	Alcohol																												
69	trans-β-Terpineol	$C_{10}H_{18}O$	1029			—	0.06	—	—	—	—	—	—	—	—	—	—	—	—	—	—	—	—	—	—	—	—	—	—
70	cis-4-Thujanol	$C_{10}H_{18}O$	1032			0.14	—	0.17	0.22	0.45	0.18	0.14	0.11	0.16	0.14	0.09	0.14	0.21	0.18	0.34	—	—	0.18	0.17	—	—	—	—	—
71	Linalool	$C_{10}H_{18}$	1059	7.4	Sweet,	52.	45.	60.	57.	16.	59.	44.	60.	53.	56.	60.	51.	57.	62.	53.	65.	62.	64.	52.	60.	56.	59.	59.	60.

		O			floral scent reminiscent of baby kale and lavender	26	76	85	90	81	83	21	45	55	47	95	23	83	41	81	79	23	52	43	18	33	66	83	19
72	trans-1-Methyl-4-(1-methylethyl)-2-cyclohexen-1-ol	$C_{10}H_{18}O$	1063			—	—	—	—	—	—	—	—	—	—	0.05	—	—	—	—	—	—	—	—	—	—	—	—	0.05
73	(-)-4-Terpineol	$C_{10}H_{18}O$	1091			0.67	—	0.08	1.34	0.25	—	0.61	—	—	—	1.88	—	—	—	0.05	—	0.78	—	0.13	—	0.13	—	1.16	1.05
74	Terpinen-4-ol	$C_{10}H_{18}O$	1091		warm peppery aroma, lighter earthy and aged wood notes	0.06	0.54	0.98	0.13	2.54	1.06	—	1.49	1.18	0.84	0.18	1.12	1.37	1.25	1.15	1.19	0.09	1.05	0.89	1.61	1.12	1.32	0.10	0.14
75	L-α-Terpineol	$C_{10}H_{18}O$	1096	330	clove flavor	0.38	0.27	—	0.04	—	—	—	—	—	—	—	—	—	—	—	—	—	1.25	—	—	—	—	—	—
76	α-Terpineol	$C_{10}H_{18}O$	1096	340	Strong floral Fragrance,	—	—	—	0.70	1.37	0.69	—	—	0.61	0.44	—	—	—	—	0.71		0.55	—	0.66	—	—	—	—	0.73

					typical Clove flavor																								
77	(-)-Myrtenol	$C_{10}H_{16}O$	1098			0.04	—	—	0.05	—	0.06	—	0.07	—	—	0.06	—	—	0.08	—	0.11	0.05	—	—	0.10	—	—	—	0.06
78	myrtenol	$C_{10}H_{16}O$	1098			—	—	0.10	—	0.24	—	0.04	—	0.12	0.04	—	0.06	0.44	—	0.19	—	—	0.14	0.07	—	0.07	0.06	0.08	—
79	Geraniol	$C_{10}H_{18}O$	1098	40	It has a warm and sweet ROSE FLOWER flavor	—	0.14	—	—	—	—	—	—	0.16	—	—	—	—	—	—	—	—	—	0.12	—	—	—	—	—
80	(Z)-3,7-dimethyl-2,6-Octadien-1-ol	$C_{10}H_{18}O$	1229	300	Sweet, lemony aroma similar to that of fresh roses	0.31	0.04	0.60	0.62	0.52	0.10	0.04	0.53	0.10	0.07	0.44	0.29	0.31	0.25	0.06	0.53	0.33	0.43	0.07	0.39	0.63	0.11	0.55	0.12
81	Peruviol	$C_{15}H_{26}O$	1468			—	—	—	—	—	—	—	0.12	—	—	—	—	—	—	—	—	—	—	—	—	—	—	—	—
82	α-Elemol	$C_{15}H_{26}O$	1469			0.07	0.04	0.17	0.06	0.18	0.18	0.07	0.16	—	0.11	0.17	—	0.16	—	0.19	0.27	0.11	0.15	0.09	0.13	0.20	0.15	0.16	0.15
83	(E)-Nerolidol	$C_{15}H_{26}O$	1479			—	—	0.75	—	—	1.37	—	2.07	0.70	0.05	1.12	—	0.14	0.08	3.12	3.51	—	1.51	0.75	2.77	2.06	—	1.21	1.27
84	(Z)-Nerolidol	$C_{15}H_{26}$	1479			—	—	—	—	—	—	—	—	—	—	—	—	—	—	—	—	—	—	—	—	—	1.3	—	—

		O																							9		
85	Espatulenol	$C_{15}H_{26}O$	1484	—	—	—	—	—	—	—	—	—	—	—	—	—	—	—	0.07	—	—	—	—	—	—	—	—
86	Bulnesol	$C_{15}H_{26}O$	1606	—	—	—	—	—	0.04	—	—	—	—	—	—	—	—	—	—	—	—	—	—	—	—	—	—
87	γ-eudesmol	$C_{15}H_{26}O$	1610	—	—	0.04	—	0.14	—	—	0.03	—	—	—	—	—	—	—	—	—	—	—	—	—	—	—	—
88	8-epi-γ-eudesmol	$C_{15}H_{26}O$	1610	—	—	—	—	—	—	—	—	—	—	0.05	—	0.06	—	—	—	—	—	—	—	—	0.04	—	—
89	(+)-γ-Eudesmol	$C_{15}H_{26}O$	1622	—	—	—	—	—	—	—	0.06	—	—	0.23	—	—	—	—	—	—	—	—	—	—	—	—	0.04
90	τ-Cadinol	$C_{15}H_{26}O$	1630	0.09	0.06	—	—	0.39	—	0.06	—	—	—	—	—	—	—	—	—	—	—	—	—	—	—	—	—
91	β-Eudesmol	$C_{15}H_{26}O$	1636	—	0.03	—	—	—	—	—	—	—	—	—	—	—	—	—	—	—	—	—	0.08	—	—	0.06	—
92	α-Eudesmol	$C_{15}H_{26}O$	1637	—	—	0.16	0.10	—	0.19	—	0.22	—	—	—	—	0.26	—	—	—	—	—	—	0.08	—	—	0.05	—
93	α-Cadinol	$C_{15}H_{26}O$	1638	0.19	—	—	—	0.76	—	—	—	0.21	—	—	—	—	—	0.20	0.27	0.24	0.26	0.19	—	0.15	0.16	—	0.16
94	2,2-Dimethyl-6-methylene-cyclohexanepropanol	$C_{15}H_{26}O$	1671	—	—	—	—	—	—	—	—	—	0.03	—	—	—	—	—	—	—	—	—	—	—	—	—	—
95	Farnesol	$C_{15}H_{26}O$	1672	—	—	—	—	—	—	—	—	—	—	0.01	—	—	—	—	0.02	—	0.02	—	—	—	—	—	—
	∑			54.	46.	63.	61.	23.	63.	45.	65.	56.	58.	65.	52.	60.	64.	59.	71.	64.	69.	54.	65.	60.	62.	63.	63.

						22	93	90	15	64	70	28	19	79	20	25	83	77	25	82	88	44	51	97	34	69	88	19	97
	Aldehyde																												
96	(R)-3,7-Dimethyl-6-octenal	$C_{10}H_{18}O$	1082	25	Strong, Fresh Grass citrus flavor	—	—	—	—	—	—	—	0.05	0.04	—	—	—	—	—	—	—	—	—	—	—	—	—	—	—
97	Citronellal	$C_{10}H_{18}O$	1082	25	Strong, Fresh Grass citrus flavor	—	—	0.06	—	—	—	—	—	—	—	—	—	—	—	—	—	—	—	—	—	0.07	0.05	—	0.06
98	Myrtenal	$C_{10}H_{14}O$	1096			—	—	—	—	—	—	—	—	—	—	—	—	0.82	—	—	—	—	—	—	—	—	—	—	—
99	Decanal	$C_{10}H_{22}O$	1203	0.1	Fresh oil Fatty, fruity Aroma	0.07	0.06	0.07	0.05	0.13	0.05	0.15	—	0.04	0.06	—	0.08	0.15	—	—	—	0.04		0.04	—	—	0.06	0.04	—
100	2-Methyl-3-phenylpropanal	$C_{10}H_{12}O$	1219			—	—	—	—	—	—	—	—	—	—	—	—	0.05	—	—	—	—	—	—	—	—	—	—	—
101	α-Thujenal	$C_{10}H_{14}O$	1239			—	—	—	—	—	—	—	—	—	—	—	—	0.05	—	—	—	—	—	—	—	—	—	—	—
102	Undecanal	$C_{11}H_{22}O$	1251	5	Rose, floral, fruity and	0.03	—	0.03	—	—	—	—	—	—	—	—	0.03	0.04	—	—	—	—	—	—	—	—	—	—	0.03

103	Dodecanal	$C_{12}H_{24}O$	1251	2	sweet orange aroma. Soap scents, waxes Fragrance, Aldehyde citrus Violet Orchid	—	—	—	0.02	—	—	—	—	—	—	—	—	—	—	—	—	—	—	—	—	—	—	—	—
104	Tridecanal	$C_{13}H_{26}O$	1251			—	0.03	—	—	—	—	0.03	—	—	—	—	—	—	—	—	—	—	—	0.02	—	0.02	—	—	—
105	Pentadecanal	$C_{15}H_{30}O$	1668			0.05	—	0.03	—	—	—	—	—	—	0.02	—	0.02	0.03	—	—	—	—	—	—	—	0.02	0.03	—	0.02
106	17-Octadecenal	$C_{18}H_{34}O$	1668			—	—	—	—	—	—	—	—	—	—	0.01	—	—	—	—	—	—	—	—	—	—	—	—	—
107	Hexadecanal	$C_{16}H_{32}O$	1669			—	0.02	0.02	0.01	0.04	—	0.06	—	0.01	—	—	—	—	—	—	—	—	—	0.01	—	—	—	0.02	—
108	Tetradecanal	$C_{14}H_{28}O$	1806	60	It is greasy and iris-like aroma gas	0.03	—	—	—	—	—	—	—	—	—	—	—	—	—	—	—	—	—	—	—	—	0.03	—	—
	∑					0.18	0.11	0.23	0.08	0.17	0.05	0.25	0.05	0.09	0.08	0.01	0.12	1.14	0.00	0.00	0.00	0.04	0.00	0.07	0.00	0.11	0.17	0.07	0.11
	Ketone																												

109	(+)-β-thujone	$C_{10}H_{16}O$	1061			—	—	—	0.13	—	—	0.16	—	—	—	—	—	3.11	0.17	—	—	—	0.12	0.09	—	0.16	—	—	—
110	Thujone	$C_{10}H_{16}O$	1061			—	—	—	—	—	—	—	0.12	—	—	0.14	—	0.80	—	0.12	0.16	—	—	—	0.14	—	—	0.17	0.18
111	Chrysanthone	$C_{10}H_{16}O$	1061			—	—	0.23	—	—	—	—	0.10	—	—	—	—	—	—	—	—	—	—	—	—	—	—	—	—
112	3-methyl-6-(1-methylethyl)-2-Cyclohexen-	$C_{10}H_{16}O$	1225			0.07	—	—	0.05	0.17	—	0.10	—	—	—	—	—	—	—	0.06	0.06	—	—	—	0.07	0.14	0.06	—	—
113	2-isopropyl-5-methyl-3-Cyclohexen-1-	$C_{10}H_{16}O$	1225			—	0.05	0.06	—	—	0.09	0.03	—	0.07	0.05	0.05	0.05	—	0.15	—	0.08	—	0.08	0.05	—	—	—	—	0.11
114	Trimethylspiro [3.5]non-5-en-1-one	$C_{12}H_{18}O$	1263			—	—	—	—	—	—	—	—	—	—	—	0.23	—	—	—	0.16	—	—	—	—	—	—	—	—
115	Acetosyringone	$C_{10}H_{12}O_4$	1648			—	—	—	—	—	—	—	—	—	—	—	—	—	—	—	—	—	—	—	—	—	—	—	0.03
116	Xanthoxylin	$C_{10}H_{12}O_4$	1649			—	—	—	—	—	—	—	—	0.06	—	—	—	—	—	—	—	—	—	—	—	—	—	—	—
	∑					0.07	0.05	0.29	0.18	0.17	0.09	0.18	0.33	0.13	0.05	0.19	0.28	3.92	0.32	0.18	0.40	0.06	0.20	0.14	0.14	0.23	0.14	0.22	0.32
	Salts																												
117	Methyl isocaproate	$C_7H_{14}O_2$	836	7	Sweet and fruity Aroma, ether	—	0.09	—	—	—	—	—	—	—	—	—	—	—	—	—	—	—	—	—	—	—	—	—	—

118	Benzeneacetic acid methylester	$C_9H_{10}O_2$	1092			—	—	—	—	—	—	—	—	—	0.03	0.04	—	—	—	—	—	—	—	—	—	—	—	—	—
119	Linalool butyrate	$C_{14}H_{24}O_4$	1224			—	—	—	—	—	0.05	—	—	—	—	—	—	—	—	—	—	—	—	—	—	—	—	—	—
120	Linalyl acetate	$C_{12}H_{20}O_2$	1224			—	—	—	—	—	—	—	—	—	—	—	—	—	—	—	—	—	—	—	—	—	0.06	—	0.07
121	(S)-(-)-Citronellic acid, methylester	$C_{11}H_{20}O_2$	1231			—	—	—	—	0.07	—	—	—	0.02	—	—	—	—	—	—	—	—	—	—	—	—	—	—	—
122	Bornyl acetate	$C_{12}H_{20}O_2$	1240	75	Woody, camphoraceous, minty, cypressy, spicy and soapy aroma	—	—	—	—	0.07	—	—	0.03	—	—	—	—	—	—	—	—	—	—	—	—	—	—	—	—
123	L-Bornyl acetate	$C_{12}H_{20}O_2$	1240	75	Aroma of forest freshness	—	—	—	—	—	—	—	—	—	0.02	—	—	—	—	—	—	—	—	—	—	—	—	—	—
124	Myrtenyl acetate	$C_{12}H_{18}O_2$	1258			—	—	0.08	—	—	0.06	—	0.06	—	—	—	—	—	—	0.15	—	—	0.06	—	—	—	—	0.06	—
125	Terpinyl acetate	$C_{12}H_{20}O_2$	1270			—	—	—	—	—	—	—	—	—	—	—	—	—	—	—	—	—	—	—	—	—	0.04	—	0.04

126	Terpinyl acetate	$C_{12}H_{20}O_2$	1279			0.03	—	0.06	0.09	—	0.21	—	0.07	—	—	0.06	0.05	—	—	—	0.07	0.03	0.07	—	—	0.10	0.11	0.25	0.11
127	(R)-lavandulyl acetate	$C_{12}H_{20}O_2$	1279			—	—	—	—	—	—	—	—	—	0.03	—	—	—	—	—	—	—	—	—	—	—	—	—	—
128	lavandulyl acetate	$C_{12}H_{20}O_2$	1290			—	—	—	—	—	—	—	—	0.13	—	0.09	—	—	—	—	—	—	0.18	—	0.11	—	—	—	—
129	Geranyl acetate	$C_{12}H_{20}O_2$	1290	9	Aroma of rose, bergamot and lavender	—	—	—	0.18	—	—	—	—	—	—	—	—	—	—	—	—	—	—	—	—	0.18	0.19	—	0.20
130	Geranyl isobutyrate	$C_{14}H_2O_2$	1290	10	Pale rose aroma and sweet apricot aroma	—	—	—	—	—	—	—	—	—	—	—	—	—	—	0.08	0.13	—	—	—	—	—	—	—	—
131	Methyl (9E)-9-octadecenoate	$C_{19}H_{36}O_2$	1675			0.03	—	0.06	—	—	—	—	—	—	—	—	—	0.08	—	—	—	—	—	—	—	—	—	—	—
132	Methyl-14-methylpentadecanoate	$C_{17}H_{34}O_2$	1856			0.06	—	—	—	—	—	—	—	—	—	—	—	—	—	—	—	—	—	—	—	—	—	—	—
133	Hexadecanoic acid methylester	$C_{17}H_{34}O_2$	1856			—	0.03	0.07	0.02	0.07	0.03	0.03	0.03	0.04	0.04	0.05	—	0.10	0.09	0.06	0.07	0.03	0.02	0.04	—	0.03	0.04	0.03	0.04
	∑					0.1	0.1	0.2	0.2	0.2	0.3	0.0	0.3	0.0	0.1	0.2	0.0	0.1	0.0	0.2	0.2	0.0	0.3	0.0	0.1	0.3	0.4	0.3	0.4

						2	2	7	9	2	5	3	2	6	2	3	5	8	9	9	7	6	3	4	1	1	3	5	6
	Acids																												
134	6-Octadecenoic acid	$C_{18}H_{34}O_2$	1691			—	—	—	—	—	—	—	—	—	—	—	—	0.10	—	—	—	—	—	—	—	—	—	—	—
135	n-Hexadecanoic acid	$C_{16}H_{32}O_2$	1877	10000	Slightly fatty, waxy aroma	—	—	—	—	—	—	—	—	—	—	—	—	0.05	—	—	—	—	—	—	—	—	—	—	—
	∑					0.00	0.00	0.00	0.00	0.00	0.00	0.00	0.00	0.00	0.00	0.00	0.00	0.15	0.00	0.00	0.00	0.00	0.00	0.00	0.00	0.00	0.00	0.00	0.00
	Olefin																												
136	(Z)-7-Hexadecene	$C_{16}H_{32}$	1486			0.04	—	—	—	—	—	—	—	—	—	—	—	—	—	—	—	—	—	—	—	—	—	—	—
137	8-Heptadecene	$C_{17}H_{34}$	1651			0.04	—	—	—	—	—	—	—	—	0.05	—	—	—	—	—	—	—	—	—	—	—	—	—	—
	∑					0.08	0.00	0.00	0.00	0.00	0.00	0.00	0.00	0.00	0.05	0.00	0.00	0.00	0.00	0.00	0.00	0.00	0.00	0.00	0.00	0.00	0.00	0.00	0.00
	Alkane																												
138	3,8-Dimethyldecne	$C_{12}H_{26}$	1232			0.03	—	—	—	—	—	—	—	—	—	—	—	—	—	—	—	—	—	—	—	—	—	—	—
139	Tetradecane	$C_{14}H_{30}$	1298			0.05	0.03	0.03	0.03	—	—	0.04	0.03	0.03	0.05	0.03	0.04	—	—	—	—	—	—	0.03	—	—	0.04	—	0.03
140	2-Methyl tetradecane	$C_{15}H_{32}$	1426			0.04	—	—	—	—	—	—	—	—	—	—	—	—	—	—	—	—	—	—	—	—	—	—	—

141	10-Methyl-eicosane	$C_{21}H_{44}$	1426	—	—	—	—	—	—	—	—	—	0.04	—	—	—	—	—	—	—	—	—	—	—	—	—	—
142	Pentadecane	$C_{15}H_{32}$	1444	0.11	—	—	—	—	—	0.12	—	—	0.22	0.10	—	0.09	—	—	—	—	—	—	—	—	—	—	—
143	Hexadecane	$C_{16}H_{34}$	1499	0.11	0.06	0.05	0.05	0.16	0.06	0.07	0.07	0.06	0.10	0.10	0.06	0.09	0.08	—	0.06	0.09	0.06	0.06	—	0.05	0.09	0.08	0.05
144	2-Methyl hexadecane	$C_{17}H_{36}$	1645	0.05	0.02	—	—	0.04	—	0.02	—	—	0.04	0.02	0.02	—	0.03	—	—	0.03	—	0.01	—	—	0.02	—	—
	∑			0.39	0.10	0.08	0.08	0.20	0.06	0.24	0.10	0.09	0.46	0.27	0.11	0.21	0.11	0.00	0.06	0.12	0.06	0.10	0.00	0.05	0.16	0.08	0.08
	Other																										
145	Heptadecyl oxirane	$C_{19}H_{38}O$	1251	—	—	—	—	—	—	—	—	—	0.02	—	—	—	—	—	—	—	—	—	—	—	0.03	—	—
	∑			0.00	0.00	0.00	0.00	0.00	0.00	0.00	0.00	0.00	0.02	0.00	0.00	0.00	0.00	0.00	0.00	0.00	0.00	0.00	0.00	0.00	0.03	0.00	0.00
