# Supplementary material for: Chemical Composition Variation in Essential Oil and Their Correlation with Climate Factors in Chinese Prickly Ash Peels (Zanthoxylum armatum DC.) from Different Habitats
Source: Molecules. 2024 Mar 18;29(6):1343. doi: 10.3390/molecules29061343 (PMC10974008; doi:10.3390/molecules29061343)
Supplement: Supplementary file 1 [file molecules-29-01343-s001.zip › Table S3.pdf]

Table S3 Relative amounts of nine major flavor compounds in each sample.

compound	Z1	Z2	Z3	Z4	Z5	Z6	Z7	Z8	Z9	Z10	Z11	Z12	Z13	Z14	Z15	Z16	Z17	Z18	Z19	Z20	Z21	Z22	Z23	Z24
Linalool	52.26	45.76	60.85	57.9	16.81	59.83	44.21	60.45	53.55	56.47	60.95	51.23	57.83	62.41	53.81	65.79	62.23	64.52	52.43	60.18	56.33	59.66	59.83	60.19
d-Limonene	19.78	22.97	15.29	14.71	29.38	14.46	22.85	13.95	17.27	14.91	14.09	20.67	11.23	19.17	18.81	10.77	13.34	11.37	19.11	14.91	16.11	15.05	15.12	16.29
Caryophyllene	1.56	1.31	0.82	0.71	3.43	1.05	1.24	1.05	1.7	1.66	1.11	1.16	0.94	0.86	1.36	1.16	1.8	1.32	1.56	1.07	0.86	0.97	1.05	0.92
Decanal	0.07	0.06	0.07	0.05	0.13	0.05	0.15	0	0.04	0.06	0	0.08	0.15	0	0	0	0.04	0	0.04	0	0	0.06	0.04	0
α-Copaene	0.05	0	0	0	0.08	0	0	0.03	0.03	0	0	0	0	0	0	0	0	0.24	0	0	0	0	0	0
Dodecanal	0	0	0	0.02	0	0	0	0	0	0	0	0	0	0	0	0	0	0	0	0	0	0	0	0
Methyl isocaproate	0	0.09	0	0	0	0	0	0	0	0	0	0	0	0	0	0	0	0	0	0	0	0	0	0
Geranyl acetate	0	0	0	0.18	0	0	0	0	0	0	0	0	0	0	0	0	0	0	0	0	0.18	0.19	0	0.2
Geranyl isobutyrate	0	0	0	0	0	0	0	0	0	0	0	0	0	0	0.08	0.13	0	0	0	0	0	0	0	0
