# Supplementary material for: Chemical Composition Variation in Essential Oil and Their Correlation with Climate Factors in Chinese Prickly Ash Peels (Zanthoxylum armatum DC.) from Different Habitats
Source: Molecules. 2024 Mar 18;29(6):1343. doi: 10.3390/molecules29061343 (PMC10974008; doi:10.3390/molecules29061343)
Supplement: Supplementary file 1 [file molecules-29-01343-s001.zip › Table S4.pdf]

Table S4 Results of person correlation analysis between volatile components and climatic factors.

Compound name	Mean annual temperature (MAT) (℃)	Mean annual minimum temperature (MAMIT) (℃)	Mean annual maximum temperature (MAMAT) (℃)	Mean annual humidity (MAH) (%)	Annual precipitation (AP) (mm)	Annual sunshine time (AST) (h)	Mean wind speed (MWS) (m/s)
Linalool	0.005	-0.010	-0.027	-0.059	-0.532**	0.402	0.084
d-Limonene	-0.107	-0.094	-0.034	0.007	0.494*	-0.376	-0.075
Caryophyllene	0.183	0.198	0.078	0.344	0.532**	-0.405*	-0.082
Decanal	-0.283	-0.222	-0.164	0.003	0.450*	-0.062	0.079
α-Copaene	0.092	0.129	0.027	0.225	0.223	-0.143	-0.121
Dodecanal	-0.046	-0.055	0.064	-0.385	-0.249	0.179	-0.218
Methyl isocaproate	-0.130	-0.066	-0.098	-0.236	-0.147	-0.240	0.044
Geranyl acetate	0.059	0.005	0.252	-0.582**	-0.340	-0.016	-0.360
Geranyl isobutyrate	0.004	-0.077	-0.350	0.227	-0.004	-0.096	0.489*

*Significant correlation at the 0.05 level (two-tailed).

**. Significant at the 0.01 level (two-tailed).
