# Supplementary material for: Chemical Composition Variation in Essential Oil and Their Correlation with Climate Factors in Chinese Prickly Ash Peels (Zanthoxylum armatum DC.) from Different Habitats
Source: Molecules. 2024 Mar 18;29(6):1343. doi: 10.3390/molecules29061343 (PMC10974008; doi:10.3390/molecules29061343)
Supplement: Supplementary file 1 [file molecules-29-01343-s001.zip › Table S5.pdf]

Table S5 Climate data averaged over the last 5 years (2017-2022) for Chinese prickly ash producing areas in China.

Place of origin (province, city/state, county/district)	Mean annual temperature (MAT) (℃)	Mean annual minimum temperature (MAMIT) (℃)	Mean annual maximum temperature (MAMAT) (℃)	Mean annual humidity (MAH) (%)	Annual precipitation (AP) (mm)	Annual sunshine time (AST) (h)	Mean wind speed (MWS) (m/s)
Sichuan, Ya'an, Tianquan	16.9	13.6	22.3	84.0	1796.5	850.0	1.2
Sichuan, Ya'an, Yucheng District	17.6	14.6	22.7	71.0	837.5	980.6	2.2
Sichuan, Liangshan, Jinyang	18.3	14.7	24.3	68	707	1328.8	1
Sichuan, Meishan, Hongya	18.8	15.5	23.5	79	1671.1	964.7	2.1
Sichuan, Meishan, Renshou	20.3	17	24.9	75	1094.3	934.2	1.4
Sichuan, Chengdu, Jintang	19.4	16	24.4	77	1314.4	957.8	1.8
Sichuan, Zigong, Yantan District	20.5	17.2	26	77	801.2	1260.0	1.7
Sichuan, Mianyang, Santai	19.3	15.6	24.4	72	942.5	1179.8	1.9
Sichuan, Zigong, Fushun	20.6	17.5	25.2	78	1007.7	1174.2	1.7
Sichuan, Guangyuan, Zhaohua District	18.2	14	24.1	69	1046.2	1389.1	1.3
Sichuan, Guang'an, Qianfeng District	19.4	16	24.7	80	918.7	1225.9	1.3
Sichuan, Guang'an, Yuechi	19.5	16.4	23.9	78	988.4	1185.8	1.6
Sichuan, Dazhou, Quxian	19.9	16.3	25.3	81	998.1	1184.6	1.4
Sichuan, Bazhong, Pingchang	19.1	15.4	25	71	858.7	1124.9	1.3
Yunnan, Zhaotong, Yanshan	12.2	7.4	16.4	74	678.0	1528.9	4.1
Guizhou, Qianxinan, Zhenfeng	16.8	14.3	21.8	79	1278.0	1492.2	3.8
Chongqing, Jiangjin	18.7	14.7	21.2	79	1022.0	1123.0	3.6
